# Levels and predictors of empathy, self-awareness, and perceived stress among nursing students: a cross sectional study

**DOI:** 10.1186/s12912-024-01774-7

**Published:** 2024-02-20

**Authors:** Shaher H. Hamaideh, Sawsan Abuhammad, Abdallah Abu Khait, Hanan Al-Modallal, Ayman M Hamdan-Mansour, Rami Masa’deh, Saleem Alrjoub

**Affiliations:** 1 Community and Mental Health Nursing Department, Faculty of Nursing, The Hashemite University, 13133 Zarqa, P.O. Box 330127, Jordan; 2 Department of Maternal and Child Health, Faculty of Nursing, Jordan University of Science and Technology, IRBID, Jordan; 3 Community Health Department, School of Nursing, The University of Jordan, Amman, Jordan; 4 School of Nursing, Applied Science Private University, Amman, Jordan

**Keywords:** Empathy, Self-awareness, Perceived stress, Nursing students, Jordan

## Abstract

**Background:**

High levels of empathy and self-awareness and low level of stress among nursing students are the core elements of improving patients’ care and outcomes. The purpose of this study is to assess the levels, relationships, and predictors of empathy, self-awareness, and perceived stress in a sample of undergraduate Jordanian nursing students.

**Methods:**

A descriptive cross-sectional design was employed to collect data from 330 students using a web-based survey.

**Results:**

The total mean scores of empathy, self-awareness, and perceived stress were 75.75, 40.17, and 55.65, respectively. Students who are studying in public universities, with higher GPA, who have no intention to leave nursing, and who are satisfied with nursing; reported higher level of empathy. Students with lower income level, who have no intention to leave nursing, and who are satisfied with nursing; reported higher level of self-awareness. Female students, those who sleeping 6 h and less, did not get a balanced diet or perform exercise, studying in public universities, lower GPA, have intention to leave nursing, and did not satisfy with nursing; reported higher level of perceived stress.

**Conclusions:**

Nursing students reported adequate empathy level, low self-awareness level, and moderate perceived stress level. Students who are satisfied and have no intention to leave nursing reported higher level of empathy, self-awareness, and perceived stress. It is necessary to conduct interventional programs that enhance students’ well-being especially empathy and self-awareness, and reduce the level of their stress.

## Background

Nurses and nursing students provides care for patients and families. Hence, possessing adequate levels of empathy and self-awareness and maintained low perceived stress level are vital components in ensuring high-quality care. Also, nurses and nursing students guide patients and their families to actively participate in health behaviors and solve their potential health problems. Therefore, high levels of empathy and self-awareness and low levels of perceived stress are the core elements of improving patients’ care and outcomes [1].

Empathy defined as ‘the ability to understand and view the world from other people’s perspectives and to connect to their experiences and feelings [2]. It is the individuals’ ability to understand and comprehend the perspectives and emotions of others in their circumstances. In essence, empathy is a subjective identification of another individual’s cognitive and affective states [3]. Examples of empathetic behaviors includes: caring for patients and their families with a genuine desire to assist them, knowing how patients and their families think out, and making oneself similar to patients and their families [2]. Empathy also is a crucial component in nurse-patient interaction [4], and increase nursing students’ emotional intelligence, compassion and quality of life [5, 6]. Empathy levels affected by many variables such as age, gender, training and experience, and students’ year of study in nursing [7, 8].

Empathy was extensively measured using Kiersma-Chen Empathy Scale (KCES) among undergraduate health professional students. For example, level of empathy among pharmacy students in Ohio, USA was 83.6 [9], and around 83.7 among pharmacy students in North Dakota, USA [10]. Several studies measured the empathy level among nursing students using different measurement scales [11–13], however, very few studies assessed empathy among nursing students using Kiersma-Chen scale [1, 3, 14–16]. For example, empathy level among nursing students were 73.4, 75.53, and 89.16 among US, Australian, and Midwest US nursing students, respectively [1, 14, 15]. Further, it was noticed that empathy decline among nursing students from beginning years to graduation years [13, 17]. Using Jefferson empathy scale, empathy was measured among Jordanian nursing students. Results found that Empathy level was lower than what have been reported in similar studies [18].

Higher levels of empathy among nursing students leads to improved patient outcomes, and increase compliance with health regimen [19]. Therefore, some researchers believe that nursing students can be prepared to be empathetic [14, 15]. Empathy is a skilled behavior that can be learned and improved by education and practice [7]. For example, using Kiersma-Chen Scale, Heidke and colleagues (2018) conducted a study to determine if integration of consumer lives experience into the content of nursing students’ courses influence the level of empathy [15]. Researchers found that empathy level was significantly increased after that integration (from 75.53 to 86.91). Also, Chen’s study (2015) found that nursing students who participated in a geriatric medication game reported a significantly increase in the level of empathy after participation in the intervention (from 89.16 to 90.91). Further, empathy was measured before and after conducting an educational program about empathy among pharmacy students. Results showed a significantly increased in empathy level after the intervention [10, 20].

Self-awareness is the use of self to analyze and guide others’ behaviors in a good way. Self-awareness enable nursing students to create a therapeutic environment with patients and encourage them to actively engage in healthy behaviors. Adequate level of self-awareness among nursing students promote high levels of empathy and self-efficacy, and decrease the level of stress and burnout. Self-awareness is the one’s tendency to pay attention to one’s emotions and behaviors in responses to a specific situation. It is a cognitive exploration of own thoughts, values, behaviors, and the feedback from others [21]. Self-awareness gives the person a clear perception about self, abilities, strengths, weaknesses, believes, thoughts, and it allows to understand others. Self-awareness is a recognition of oneself as a social person affecting others, recognition of others’ concerns, and recognition of self-anxiety as a social person [21].. Self-awareness is a dynamic process aimed toward providing better care, and it is important in developing a therapeutic relationship with clients and their families [22]. Many studies measured self-awareness among nursing students [23, 24], however, very few studies used self-consciousness scale [25]. For example, among US nursing students, self-awareness level using self-consciousness scale was 53.7 [1].

Perceived stress is an important issue among most university students especially nursing students [26]. Nursing is considered a stressful profession. Further, studying nursing is also considered as stressful due to high educational and clinical demands. High levels of stress among nursing students were associated with lower quality of life and well-being, lower educational performance, and higher psychological disturbances. Stress is perceived when the person appraised the demands as exceeding his or her resources and abilities. Lazarus and Folkman (1984) defined stress as “a particular relationship between the person and his/her environment, that is appraised as exceeding the available resources and enduring the well-being” [27]. Several studies conducted to measure the levels of perceived stress among nursing students. For example, using PSS-10, the levels of perceived stress were 20.94 and 21. 54 among UK, and Chinese nursing students, respectively [28]. In most studies, nursing students reported moderate level of perceived stress. For example, among Moroccan nursing students, stress levels were 21.70%, 59.10%, and 19.20 for low, moderate, and high levels, respectively [29]. In addition, a study conducted in Egypt found that 90% of nursing students reported a moderate level of stress, and found significant relationship of stress with demographic variables [30].

### Significance and research questions

Through their entire nursing study, university nursing students usually engage with patients and their families which potentially leading to increased their stress levels. Therefore, during clinical training, nursing students have to have good levels of empathy and self-awareness which may decrease their level of perceived stress. Several studies explored levels and predictors of empathy, self-awareness, and perceived stress among nurses. For example, Abu Lebda and colleagues (2023) measured self-awareness, empathy, and patient centered-care among Jordanian nurses. They found that nurses had higher level of self-awareness and empathy, and low level of patient-centered care [31]. Also, few studies assessed the levels of empathy, self-awareness, and perceived stress among nursing students. However, there is scarcity in research studies that assess those concepts among Jordanian university nursing students collectively, therefore, this study was undertaken. More specifically, this study aimed to:

1. Assess the levels of empathy, self-awareness, and perceived stress among Jordanian undergraduate nursing students?
2. Examine the relationships among empathy, self-awareness, and perceived stress among Jordanian undergraduate nursing students?
3. Detect any differences in empathy, self-awareness, and perceived stress with selected demographic and educational variables among Jordanian undergraduate nursing students?
4. Assess the predictor variables of empathy, self-awareness, and perceived stress among Jordanian undergraduate nursing students?

## Methods

### Design

A descriptive, cross-sectional, correlational design was used.

### Instruments

A pilot testing for the scales was done to check the feasibility and applicability of the scales using 20 nursing students [32]. No changes were required. The questionnaire consisted of four parts.

1. *Demographic and educational variables.* Demographic variables were: age, gender, family monthly income (by Jordanian Dinar), average daily sleeping hours, smoking status (no, yes), taking well-balanced diet (no, yes-if eating a wide variety of foods from all components including water), and practicing exercise regularly (no, yes-if performing at least 30 min of exercise for 3 times per week). Educational variables were: university type (public, private), Grade Point Average (GPA: excellent, very good, good, or fair), university academic year (second, third, or fourth), intention to leave nursing study (no, yes), and level of satisfaction with nursing study (scale from 1 to 5).
2. *Kiersma-Chen Empathy Scale (KCES)* was used to assess the level of empathy among nursing students. KCES consists of 15 items on a Likert-type scale ranging from “1” indicating (strongly disagree) to “7” indicating (strongly agree). The total score for the scale ranging from 15–105, with higher score, indicating higher empathy (3). KCES composed of two subscales: cognitive domain (9 items) and affective domain (6 items). It also has good validity and reliability. Cronbach’s alpha was 0.80 among nursing students [3]. In the current study, the Cronbach’ s alpha was 0.79.
3. *Self-Consciousness Scale-Revised (SCS-R)* was used to measure self-awareness among nursing students [29]. The scale is consisted of 22 items grouped into 3 subscales: private self-consciousness (9 items), public self-consciousness (7 items), and social anxiety (6 items). SCS-R is a Likert-type scale that range from 0 indicating (not like me at all) to 3 indicating (a lot like me). The possible score for the scale range from 0–66, with higher score, indicating the higher the level of self-awareness. SCS-R has good validity and reliability. Cronbach’s alpha was 0.73, and test-retest was 0.89 [33, 34]. In the current study, the Cronbach’ s alpha was 0.84.
4. *Perceived Stress Scale (PSS)* was used to assess the levels of perceived stress among nursing students during the last month. PSS is the most widely used tool to measure the perceived stress across different countries, cultures, and age groups including university students. PSS consists of 10 items with responses ranging from “0” indicating “never” to “4” indicating “very often”. The total score range from 0 to 40, with higher scores indicating higher perceived stress. A score between 0 and 13 indicating low level, between 14 and 27 indicating a moderate level, and between 28 and 40 indicating high level [35]. PSS has good validity and reliability. In the current study, the internal consistency reliability as measured by Cronbach’s alpha was 0.81.

### Sample

G*Power software was used to estimate the required sample size. For linear multiple regression with 13 predictor variables, and using a medium effect size (0.15), a power of 0.80, an alpha of 0.05, and a two-tailed test, the minimum required sample size is about 131 participants [36]. All full-time Jordanian undergraduate nursing students who are enrolled in any Jordanian university during the second semester of the academic year 2021–2022 were invited to participate in the study. Non-Jordanians, graduates, and part-time students were excluded.

### Setting

There are 14 Jordanian universities (6 are public and 8 are private) that offer a Bachelor’s degree in nursing with around 4000 students. Undergraduate nursing programs consisted on average of 132 credit hours distributed into 4 years. During first year, nursing students study general sciences, anatomy, physiology, and introductory nursing courses. Starting from the second year, students start to study both theoretical and clinical courses. Clinical courses were taken in clinical areas where they provide care (help) to the patients and their families. During second year, they study adult nursing. During third year, they study maternity and pediatric nursing. During fourth year, they study community and psychiatric nursing, management, and professional training.

### Data collection

Data were collected from a convenience sample through a web-based survey during the period of March 2022 by uploading the web-based link survey. Interested and eligible students were asked to take part in the study voluntarily. In the first page of the survey, an informed consent was obtained from by asking students about their willingness and consent to participate in the study. An online invitation to participate was sent to nursing students’ groups in each Jordanian university via Google forms. The online survey was posted on colleges of nursing groups of official portals and social media pages (Facebook, Moodle, Microsoft teams). In the online invitation letter, only nursing students were asked to respond to the questionnaire.

### Ethical considerations

The study’s ethical approval has been approved by the Institutional Review Board (IRB) of the Hashemite University (HU) where most of the researchers are working (No: 10-1-2021-2022). IRB at HU depends on the Declarations of Helsinki. In introduction, the participants told about study’s purposes and that their participation is voluntary, and their data confidentiality is protected. The introduction also contained a statement regarding the informed consent. To promote the participants’ confidentiality, no personal identification data were collected.

### Data analyses

The data were analyzed using the Statistical Package for Social Sciences (SPSS-version 25) program. Descriptive statistics were used to describe sample characteristics and the levels of empathy, self-awareness, and perceived stress. Pearson’s product-moment was used to measure the correlations among variables. Independent sample t-test and ANOVA were used to examine the differences in the mean scores between demographics and study variables. General regression analyses were performed separately to detect the predictor variables of empathy, self-awareness, and perceived stress. A significant *p*-value set as of 0.05. All regression assumptions were met.

## Results

### Demographic variables

The sample consisted of 330 Jordanian undergraduate nursing students, around two thirds of them were females (*n* = 214, 64.8%). Their age ranged from 18 to 33 years (mean = 20.61, SD = 2.31). Three fourth of the students were studying at public university (*n* = 253, 76.7%). Few students (*n* = 46, 13.6%) reported their intention to leave nursing studying, and around 44 (13.4%) reported that they are not satisfied with nursing studying. Other demographic variables were shown in Table 1.

**Table 1:** Demographic variables (*N* = 330)

Variable (Range)	Mean	Standard deviation
**Age**	20.61	2.31
**Monthly family income (Jordanian Dinar)**	768.7	767.3
**Average sleeping hours/day**	6.74	1.54
	Frequency	Percentage
**Gender**		
Male	116	35.2
Female	214	64.8
**Smoking status**		
No	251	76.1
Yes	79	23.9
**Eating a well-balanced diet**		
No	202	61.2
Yes	128	38.8
**Performing physical exercise regularly**		
No	261	79.1
Yes	69	20.9
**University Type**		
Public	253	76.7
Private	77	23.3
**GPA**		
Fair	5	1.5
Good	56	17.0
Very good	147	44.5
Excellent	122	37.0
**University academic year**		
First year	63	19.1
Second year	96	29.1
Third year	62	18.8
Fourth year	109	33.0
**Do you think to leave nursing study?**		
No	284	86.1
Yes	46	13.9
**Satisfaction with nursing study?**		
Strongly not satisfied	21	6.4
Moderately not satisfied	23	7.0
Neutral	42	12.7
Moderately satisfied	126	38.2
Strongly satisfied	118	35.8

#### Levels of empathy, self-awareness, and perceived stress

The total mean score of empathy was 75.75 (SD = 9.66). When dividing the mean at the number of items (15 items), empathy average was 5.05 out of 7 (SD = 0.64). In regard to empathy domains (subscales), the affective domain (mean = 5.33, SD = 0.84) was higher than the cognitive one (mean = 4.86, SD = 0.66). The highest item in empathy scale was “It is necessary for a healthcare practitioner to be able to value someone else’s point of view” (mean = 5.72, SD = 1.26). However, the lowest item was " I will not allow myself to be influenced by someone’s feeling when determining the best treatment” (mean = 2.76, SD = 1.57). (See Table 2).

**Table 2:** Mean scores of empathy, self-awareness, and perceived stress (*N* = 330)

Scale (Possible score)	Number of items	Mean	SD	Mean/No. of items (SD)
Empathy (15–105)	15	75.75	9.66	5.05 (0.64)
Cognitive domain (9–63)	9	43.75	6.0	4.86 (0.66)
Affective domain (6–42)	6	32.00	5.03	5.33 (0.84)
Self-awareness (0–66)	22	40.17	8.94	1.83 (0.41)
Private self-consciousness (0–27)	9	18.79	3.91	2.09 (0.43)
Public self-consciousness (0–21)	7	12.60	3.65	1.80 (0.52)
Social anxiety (0–18)	6	8.78	4.68	1.46 (0.78)
Perceived Stress (0–40)	10	22.65	6.76	2.26 (0.67)

The total mean score of self-awareness was 40.17 (SD = 8.94). When dividing the total mean at the number of items (22 items), self-awareness average was 1.83 out of 3 (SD = 0.41). In regard to empathy domains (subscales), the highest score was for private self (mean = 2.09, SD = 0.43), and the lowest was the social anxiety domain (mean = 1.46, SD = 0.78). The highest item in self-awareness was “I am always trying to figure myself out” (mean = 2.44, SD = 0.75). The lowest item was “I am self-conscious about the way I look” (m = 0.88, SD = 1.06).

The total mean score of perceived stress was 22.65 (SD = 6.76). Thirty-four students (10.3%) reported low level of stress, 197 (59.7%) reported moderate level of stress, and 99 (30.0%) reported high level of stress. When dividing the total mean at the number of items (10 items), perceived stress average was 2.26 out of 4 (SD = 0.67). The highest item in perceived stress was “In the last month, have often have you felt nervous and stressed” (mean = 2.82, SD = 1.20). The lowest item was “In the last month, have often have you felt confident about your ability to handle your personal problems” (mean = 1.61, SD = 1.12).

#### Correlations of empathy, self-awareness, and perceived stress with demographics

A significant positive correlation was found between self-awareness and perceived stress (0.196). However, empathy did not correlate with self-awareness or perceived stress. Empathy correlated positively with satisfaction in nursing studying. Self-awareness correlated positively with perceived stress and intention to leave nursing. Perceived stress correlated positively with intention to leave nursing, and negatively with satisfaction in nursing. (See Table 3).

**Table 3:** Correlations of empathy, self-awareness, perceived stress, and demographics (*N* = 330)

Variable	Empathy	Self-awareness	Perceived Stress
Empathy	1		
Self-awareness	0.046	1	
Perceived stress	-0.008	0.196**	1
Sleeping hours	-0.035	0.007	-0.165**
Gender	0.072	0.047	0.231**
Smoking status	-0.120*	-0.011	-0.026
Taking Well-balanced Diet	0.006	-0.054	-0.249**
Performing Exercise	-0.031	-0.026	-0.195**
Type of University	-0.119	-0.092	-0.109*
Grade Point Average	0.147**	-0.061	-0.112*
Intention to Leave nursing	-0.094	0.155**	0.191**
Satisfaction in nursing	0.152**	-0.092	-0.295**

* Significance at alpha of 0.05 two-tailed

** Significance at alpha of 0.001 two-tailed

#### Differences in empathy, self-awareness, and perceived stress according to demographics

Regarding empathy; significant differences were found in relation to university type, GPA, intention for leaving nursing studying, and satisfaction with nursing studying. Students in public universities, with higher GPA, who have no intention to leave nursing, and those who satisfied with nursing reported higher level of empathy.

Regarding self-awareness; significant differences were found in relation to income level, intention for leaving nursing studying, and satisfaction with nursing studying. Students lower income level, who have no intention to leave nursing, and those who satisfied with nursing reported higher level of self-awareness. Regarding perceived stress; significant differences were found in gender, sleeping hours, taking well-balanced diet, performing physical exercise, university type, GPA, intention for leaving nursing studying, and satisfaction with nursing studying. Female students, sleeping less, who did not get a well-balanced diet or performing exercise, in public universities, with lower GPA, who have intention to leave nursing, and those who did not satisfied with nursing reported higher level of empathy. (See Table 4).

**Table 4:** Differences of empathy, self-awareness, and perceived stress with demographics

Variable	Empathy		Self-awareness		Perceived stress	
	Mean (SD)	*p*	Mean (SD)	*p*	Mean (SD)	*p*
**Gender**						
Male	74.97	0.194	39.51	0.397	20.58	**> 0.001**
Female	76.16		40.52		23.77	
**Age**						
18–20	76.14	0.400	40.64	0.258	22.72	0.822
21–33	75.24		39.58		22.55	
**Income level**						
0-500 JD	75.80	0.909	41.38	**0.010**	23.03	0.288
501 JD and more	75.68		38.84		22.23	
**Sleeping hours**		0.087				
6 or less	76.67		40.89	0.150	23.88	> **0.001**
7 and more	74.85		39.47		21.46	
**Smoking status**						
No	76.28	0.072	40.19	0.397	22.72	0.711
Yes	74.03		40.10		22.41	
**Balanced diet**						
No	75.81	0.843	40.43	0.331	23.98	> **0.001**
Yes	75.64		39.77		20.55	
**Physical exercise**						
No	75.85	0.578	40.22	0.637	22.26	> **0.001**
Yes	75.32		39.69		20.32	
**University type**						
Public	76.26	**0.030**	40.64	0.095	23.05	**0.049**
Private	74.05		38.62		21.32	
**GPA**						
Fair/Good	73.33	**0.011**	41.51	0.219	24.35	**0.033**
Very good	75.46		39.68		22.41	
Excellent	77.46		39.86		21.94	
**Academic Year**						
First year	76.41	0.724	41.12	0.334	21.85	0.772
Second year	76.06		41.01		22.96	
Third year	75.46		39.63		23.08	
Fourth year	75.06		39.62		22.58	
**Leaving nursing studying**						
No	76.23	**0.021**	39.70	**0.005**	22.13	> **0.001**
Yes	72.69		43.06		25.85	
**Satisfaction**						
Not satisfied	72.43	**0.041**	42.05	**0.016**	26.43	> **0.000**
Satisfied	76.58		39.68		21.69	

#### Predictors of empathy, self-awareness, and perceived stress

Three separate regression analyses were performed to detect the predictors of empathy, self-awareness, and perceived stress. All sociodemographic variables were entered as Independent variables. Regarding empathy, two factors (GPA and Satisfaction in nursing studying) predicting empathy and accounted for 4% of the total variance. An increase in GPA score by one will increase significantly the total score of empathy by 0.153. Also, an increase is satisfaction in nursing studying will increase the total score of empathy by0.131. Concerning self-awareness, one factor, which is perceived stress, predicted self-awareness and accounted for 3.6% of the total variance. An increase in perceived stress by one will increase significantly the total score of self-awareness by 0.169. Regarding perceived stress, five factors (Satisfaction in nursing studying, gender, eating well-balanced diet, self-awareness, and sleeping hours) predicted perceived stress and accounted for 21% of the total variance. An increase in satisfaction in nursing studying will decrease significantly the perceived stress by -0.264. Also, not eating a well-balanced diet and a decrease in sleeping hours will increase significantly the perceived stress by -0.179 and − 0.133, respectively. Being male and having elevated level of self-awareness will increase the level of stress by 0.155. (See Table 5).

**Table 5:** Stepwise multiple regression model predicting empathy, self-awareness, and perceived stress (*N* = 330)

Predictors of empathy	B	β	Adj. R^2^	*F*	*p*	95% CI			
						Lower	Upper		
1. Grade Point Average	1.957	0.153	0.026	9.913	0.002	0.587	3.327		
2. Satisfaction in studying nursing	1.095	0.131	0.040	7.897	> 0.001	0.196	1.994		
**Model Summary**									
R^2^	0.046								
Adjuster R^2^	0.040								
Total Variance	4%								
**Predictors of self-awareness**									
1. Perceived stress	0.260	0.169	0.036	13.148	> 0.001	0.119	0.400		
**Model Summary**									
R^2^	0.039								
Adjuster R^2^	0.036								
Total Variance	3.6%								
**Predictors of perceived stress**									
1. Satisfaction in studying nursing	-1.550	-0.264	0.084	31.20	> 0.001	-2.122	-0.978		
2. Gender	3.005	0.212	0.137	27.15	> 0.001	1.635	4.375		
3. Eating well-balanced diet	-2.486	-0.179	0.174	24.17	> 0.001	-3.847	-1.124		
4. Self-awareness	0.117	0.155	0.195	20.97	> 0.001	0.044	0.190		
5. Sleeping hours	-0.584	-0.133	0.210	18.54	> 0.001	-1.012	-0.156		
**Model Summary**									
R^2^	0.222								
Adjuster R^2^	0.210								
Total Variance	21%								

* Significance at alpha of 0.05 two-tailed

CI: Confidence Interval for B.

## Discussion

Results of this research study found that the total mean score of empathy was 75.75 (SD = 9.66). This result is within the reported range of empathy level among nursing students. For example, empathy level was 73.4 and 75.53 among US and Australian nursing students [1, 14], which are lower than levels found among pharmacy students with 83.6 and 83.7 [9, 10], respectively. However, literature indicating that empathy level can be increased and improved by education and training. In this regard, US nursing students’ empathy level was significantly improved after participating in a medication game program that is directed toward geriatric. Empathy level increased from 89.16 to 90.91 [14]. Also, also Australian nursing students improved their empathy level after studying the consumers’ lived experience of interview in a first year course. empathy level increased from 75.53 to 86.91 [15]. Further, results of this study were congruent with the results of a Jordanian study that measure empathy among Jordanian nursing students. They found that the reported empathy was lower than the scores reported in similar studies, and that female nursing students scored higher than male on the empathy scale [31].

Results of this research study found that the total mean score of self-awareness was 40.17 (SD = 8.94). This result is lower than what was found by Haley et al., (2017) who found the level of self-awareness among US nursing students to be 53.7. Among Iranian students, self-awareness level was found to be 50.85 [37], and among Pakistani university students was 40.0 [38]. This means that Jordanian nursing students have lower levels of self-awareness than what was found in similar few studies. Also, our results regarding self-awareness was lower than what was found among Jordanian nurses [31].

Results of this research study found that the total mean score of perceived stress was 22.65 (SD = 6.76). This result is consistent with or a little bit higher than results from most of the previous studies that measure the perceived stress among nursing students. For example, perceived stress level ranged between 18.92 [39] to 22.78 [40]. Also, the perceived stress was 21.20 among nursing students in Hong Kong [41]. Regarding the percentages of low, moderate, and high levels of perceived stress, results of this study showed that these levels were 10.3%, 59.7%, and 30.0% respectively. These results were almost within the range found in other studies, with slight increase in the percentage of high level. For example, among Indian nursing students the percentages were 13%, 83%, and 4%, respectively [39], and among Moroccan students were 21.7%, 59.1%, and 19.2%, respectively [29]. Also, among Egyptian nursing students, the level of low, moderate, and high perceived stress were 6.8%, 90.0%, and 3.2%, respectively [30].

Results of this study showed that the students in public universities, with higher GPA, have no intention to leave nursing, and who are satisfied with nursing reported higher level of empathy. Results regarding satisfaction in nursing and the intention to leave nursing were consistent with results found by Ouzouni and Nakakis (2012) who found that nursing students who are willing to work in nursing after graduation and who are satisfied with nursing displayed more empathy than those who have intention to leave nursing and work in non-nursing disciplines. Regarding GPA, results of this study were consistent with results found that students with higher GPA reported higher level of empathy [42].

Results of this study showed that the students with lower income level, do not have no intention to leave nursing studying, and who are satisfied with nursing studying reported higher level of self-awareness. Perceived stress predicted self-awareness. Contrary to what was found in pervious literature, results of this study showed no relationships between empathy and self-awareness. Contrary to what was found by Lee and Nam (2016), self-awareness and empathy in this study did not significantly associate with each other. Also, Buvaneswari and Sylvia (2018) found that self-awareness was higher among female students and among students in their first year [23]. Consistent with what was found in this study, Putri et al. (2020) found a relationship between self-awareness and perceived vulnerability and stress [43].

Results of this study showed that those female students, who sleeping less, did not get a well-balanced diet or perform exercise regularly, studying in public universities, with lower GPA, have intention to leave nursing, and did not satisfied with nursing reported higher level of perceived stress. These results were consistent with the results found in previous similar studies. For example, level of perceived stress was higher among female Spanish nursing students [44], among Hong Kong female nursing students [41], among Moroccan female nursing students [29], and among Jordanian female students [45]. However, no significant difference was found between male and female Saudi Arabian nursing students [45]. Satisfaction in nursing studying, gender, eating well-balanced diet, self-awareness, and sleeping hours predicted perceived stress. These results also congruent with pervious results which found that satisfaction with nursing decrease the level of perceived stress [38, 46].

### Limitations

Although this study provides valuable information about empathy, self-awareness, and perceived stress, it has a few limitations. First, a cross-sectional design was employed; therefore, a causal relationship cannot be drawn. Second, bias and subjectivity may be an issue due to using a self-rating questionnaire to collect data. Students may tend to over or under rate their responses to be socially desirable. Using an online questionnaire makes it challenging to generate random samples since the respondents are only those who have access to the Internet.

### Recommendations

There is a need for effective strategies to foster level of empathy and self-awareness, and to decrease the level of perceived stress among Jordanian undergraduate nursing students. Of these strategies may incorporate and integrate those concepts in the nursing curriculum explicitly or/and performing an extensive interventional programs [47]. More attention should be focused on ways to enhance and improve self-awareness among nursing students [22]. Those interventional programs should include the reflection method as part of the interventional content because this method was used mostly and showed adequate results [48]. Also, there is a need for more research studies that can identify the predictor variables of the concepts studies especially the empathy and self-awareness. Further, conducting longitudinal studies over all academic years is recommended in order to identify changes in empathy, self-awareness, and perceived stress overtime. Further, more actions that reflect the true image of social status of the nursing profession should be enhanced [49].

## Conclusions

Results of this study found that nursing students have adequate empathy level, low self-awareness level, and moderate perceived stress level. Students who are satisfied in nursing and have no intention to leave nursing reported higher level of empathy, self-awareness, and perceived stress. Nurse educators should implement interventional programs that enhance nursing students’ well-being. Examples of these programs are stress management, time management, self-awareness and self-concept. Also, more actions that reflect the true image of the social status of the nursing profession should be enhanced among nursing students and public [49].

## Data Availability

All relevant data are included with in the manuscript document. Data available on request from the authors.
